# Air Pollution Increases Risk of Occurrence of Intracerebral Haemorrhage but Not of Subarachnoid Haemorrhage: Time-Series Cross-Sectional Study

**DOI:** 10.3390/biomedicines12071562

**Published:** 2024-07-15

**Authors:** Radosław Czernych, Grzegorz Kozera, Artur Jerzy Badyda, Leszek Bieniaszewski, Paweł Zagożdżon

**Affiliations:** 1 Department of Hygiene and Epidemiology, Faculty of Medicine, Medical University of Gdańsk, 80-210 Gdańsk, Poland; pzagoz@gumed.edu.pl; 2 Centre of Medical Simulations, Medical University of Gdańsk, 80-204 Gdańsk, Poland; gkozera@gumed.edu.pl (G.K.); leszek.bieniaszewski@gumed.edu.pl (L.B.); 3 Department of Informatics and Environment Quality Research, Faculty of Building Services, Hydro- and Environmental Engineering, Warsaw University of Technology, 01-604 Warsaw, Poland; artur.badyda@pw.edu.pl

**Keywords:** air pollution, gaseous pollutants, particulate matter, haemorrhagic stroke, intracerebral stroke, subarachnoid stroke

## Abstract

(1) Background: Haemorrhagic strokes (HS), including intracerebral (ICH) and subarachnoid haemorrhages (SAH), account for approximately 10–15% of strokes worldwide but are associated with worse functional outcomes and higher rates of mortality, and financial burden than ischemic stroke. There is evidence that confirmed poor air quality may increase the incidence of haemorrhagic strokes. The aim of our study was to evaluate the association between individual ambient air pollutants and the risk of haemorrhagic stroke in an urban environment without high levels of air pollution. (2) Methods: A time-series cross-sectional study design was used. A daily air pollution concentration (Agency of Regional Air Quality Monitoring in the Gdansk Metropolitan Area) and incidence of haemorrhagic strokes (National Health Fund) were obtained and covered the time period from 1 January 2014 to 31 December 2018. A generalised additive model with Poisson regression was used to estimate the associations between 24-h mean concentrations of SO_2_, NO, NO_2_, NOx, CO, PM10, PM2.5, and O_3_ and a daily number of haemorrhagic strokes. (3) Results: The single-day lag model results showed that NO_2_, NO and NO_x_ exposure was associated with increased risk of ICH (88% events) with RR of 1.059 (95% CI: 1.015–1.105 for lag0), 1.033 (95% CI: 1.007–1.060 for lag0) and 1.031 (95% CI: 1.005–1.056 for lag0), but not for SAH (12% events). Exposure to CO was related to a substantial and statistically significant increase in incidence for 1.031 (95% CI: 1.002–1.061 for lag0) but not for SAH. Higher SO_2_, PM_10_, PM_2.5_, and O_3_ exposures were not significantly related to both ISC and SAH. (4) Conclusions: In this time-series cross-sectional study, we found strong evidence that supports the hypothesis that transient elevations in ambient NO_2_, NO and CO are associated with a higher relative risk of intracerebral but not subarachnoid haemorrhage.

## 1. Introduction

Haemorrhagic strokes (HS), including more common intracerebral (ICH) and less frequent subarachnoid haemorrhage (SAH), remain one of the major causes of mortality and disability across the globe. HS has an overall prevalence of 116.6 per 100,000 people worldwide and occurs most commonly in developed countries and in Asians [1,2]. HS accounted for approximately 10–15% of strokes worldwide but was associated with worse functional outcomes and higher rates of mortality, morbidity, and financial burden than ischemic stroke [2,3,4].

According to the Global Burden of Disease Study (2015), which analysed data across 25 years, ambient air pollution was found to have contributed substantially to diseases such as ischemic heart disease, cerebrovascular disease, chronic obstructive pulmonary disease and their resultant mortality and disability-adjusted life years loss [5]. Indeed, there is a growing body of evidence on the association between exposure to air pollution and HS [6,7,8,9]. Existing studies suggest that short-term exposure to PM_2.5_, PM_10_, and ozone was significantly associated with the risk of HS [7,10], intracerebral haemorrhage [8,11], and fatal intracerebral haemorrhage [9]. Until now, there have been at least two possible mechanisms that could explain the relationship between air pollution and ICH [12]. First, chronic exposure to air pollution is associated with hypertension, which could lead to remodelling of the intracranial arteries and increase the risk of ICH [13,14,15]. Second, air pollution may directly contribute to and promote endothelial injury and vasodilatory effects that could increase the risk of aneurysm rupture [16].

Although the above-mentioned effect of polluting agents on ICH seems evident, most studies suggest that the same pollutants do not contribute to an increase in SAH incidence [8,9]. Air pollution refers to the presence of harmful airborne substances that arise from the complex interaction between natural and anthropogenic environmental conditions [17]. Air pollution is considered a serious public health problem, and its important constituents comprise but are not limited to particulate matter of aerodynamic diameter of ≤2.5 μm (PM_2.5_) and ≤10 μm (PM_10_), ozone (O_3_), nitrogen dioxide (NO_2_), sulphur dioxide (SO_2_) and carbon monoxide (CO) [18,19]. 

While the above-mentioned studies report associations between ambient air pollutants and HS, these associations were inconsistent, and higher-quality studies are urgently needed [5,20]. Moreover, the majority of studies examining the short-term relationship between air quality and HS risk were undertaken in temperate settings [5,20]. Few studies have explored the association between ambient air pollutants and HS in a coastal setting [21,22]. Gdansk, compared with other Polish agglomerations, i.e., Warsaw or Cracow, has the lowest air pollution [23,24,25,26]. Thus, we aimed to evaluate the association between individual ambient air pollutants and the risk of HS in a coastal city in a low-air-pollution area.

## 2. Materials and Methods

### 2.1. Study Area

Gdansk is a city situated on the southern coast of the Baltic Sea in northern Poland. With a population of over 470,000 residents and covering an area of 262 km^2^, it is the capital and largest city of the Pomeranian Voivodship [27]. Due to its unique geographic location, Gdansk experiences both oceanic and continental climatic influences. The proximity to the Baltic Sea affects the speed and direction of winds within the city, making them stronger and more variable compared to inland areas of Poland. Similar to other urban areas in Poland, the green-blue spaces of the town area are less than 50% [28]. The primary source of air pollution in the Pomeranian Voivodship is anthropogenic emissions. These emissions are predominantly from industrial plants, particularly fuel combustion for energy production and technological processes (23%), transportation (road, rail, water, and air) accounting for 15%, and the municipal and housing sector, which contributes 49% of the pollution [28]. 

### 2.2. Incidence and Environmental Data

All the incidents of haemorrhagic stroke data were obtained from the National Health Fund (NHF). The HS incidence cases were clinically diagnosed by the local sentinel hospitals and were simultaneously reported to the NHF through case report cards. More than 95% of HS cases have been hospitalised in national stroke units’ networks or neurosurgery departments. Each unit had to provide a standardised clinical diagnostic and to prove, with neuroimaging, a stroke aetiology. All cases have been reported to NHF using a dedicated application form. The data set included the ID number, sex, age, date of birth, onset data of stroke, type of stroke diagnosis, ICD-10 encoding, and current address for each case. HS was divided into two-stroke subtypes (intracerebral and subarachnoid haemorrhage, I61 and I60, respectively) according to the International Classification of Diseases 10th Revision (ICD-10). The division of the analysed population was based on sex and age. Age sub-division into elderly (age ≥ 65 years) and non-elderly (age < 65 years) was based on World Health Organization (WHO) standards [29]. The calendar year was divided into summer (April–September) and heating (October–March) periods according to local climatic characteristics [27]. 

Average hourly concentrations data of chosen gaseous pollutants, namely SO_2_, NO_2_, NO, NO_x_, PM_2.5_, PM_10_, CO, and O_3_, were facilitated by the Foundation: Agency of Regional Air Quality Monitoring in the Gdansk metropolitan area (ARMAAG) and covered the timespan According to the WHO’s Air Quality Guidelines. The environmental data time structure was adjusted to incidence data according to WHO’s Air Quality Guidelines. Therefore, we used a daily maximum of 8-h-mean concentration for ozone and mean concentration of the remaining gaseous pollutants. Missing values were imputed by utilising the multiple-interpolation method [30]. Daily average meteorological data (temperature, relative humidity, and atmospheric pressure) for the same period of time were also collected from ARMAAG and served for standardisation purposes. 

### 2.3. Statistical Methods

The descriptive statistics, including mean with standard deviation (SD), minimum (Min), maximum (Max), and interquartile range (IQR), were used to describe the data on incident stroke cases, air pollutants, and meteorological factors. In order to evaluate the seasonal dependence between air pollutant emissions and stroke incidence, we calculated the above-mentioned descriptive statistics for the warm and cold seasons separately. Spearman rank correlation analysis was used to analyse the correlation between air pollutants and meteorological factors. For our time-series study, to evaluate the risk of short-term, low-level air pollutant exposure, we applied the generalised additive model (GAM) with Poisson regression. Generalised additive models are a general class of models that allow for parametric and nonparametric forms of relationship between a continuous predictor and a continuous, normally distributed outcome. This method has been used by numerous researchers [31,32,33]. In our study, the daily number of hospital admissions among Gdansk residents is rare, with a non-linear relationship between independent variables and the dependent ones (HS incidence per day). Therefore, the GAM based on Poisson distribution was used to explore the effects of air pollutants on the risk of incident stroke, in which potential confounding factors, i.e., trends, national holidays and meteorological factors [34,35,36].

The Poisson regression model is described by the equation:

(1) $$\log\left(E\left(y_{t}\right)\right)=\sum_{i=1}^{m}\beta_{i}(x_{t})+\sum_{i=m+1}^{p}s_{i}(x_{t,l})$$

In its general form, the Poisson model with lagged effects are represented by the logarithm of the expected values of the number of events (haemorrhagic strokes) *y_t_* at time *t*, which is in turn explained by a linear combination of the *i*-th functions of the predictors *p_i_* (*x_t_*) expressed by delaying related with moment *t*, and splines that express the delay with the moment *t* by a period *l*, where *l* = 0, …, *L*. *L* means the maximum delay. *β* is the regression coefficient that indicates the relative risk of stroke associated with a one-unit increase in pollutant concentration, while *x_t_* is the exposure factors (atmospheric pollutants and meteorological conditions).

We also estimated the lag effects of low-level given air pollutants on the risk of HS incidence incidents for single-day lag effects (lag0, lag1, lag2, lag3). These lag effects represented how the air pollution concentration on a given day affected health outcomes on the following days. In the single-day exposure models, lag0 indicated the air pollutant concentration on the same day, while lag1 referred to the concentration from the previous day and so forth. Based on the above models, we obtained relative risk assessments corresponding to increases in pollutant concentration levels by the interquartile range (IQR) and changes in temperature by 10 degrees Celsius, humidity by 5%, and atmospheric pressure by 5 hPa. We focused on air pollutants that had relative risks (RRs) and 95% confidence intervals for IQR changes in pollution levels, with a significance level of less than 0.05 in the single-pollutant model.

All calculations and graphs were produced using the R statistical package (version 3.4.1).

## 3. Results

### Descriptive Analysis and Correlation Analysis

Table 1 presents the results of descriptive statistics for daily incidents of haemorrhagic stroke cases in Gdansk for the years 2014–2018. During 1 January 2014 and 31 December 2018, there were a total of 5181 incident cases of HS. Out of the total number of HS cases, the SAH cases and ICH accounted for 12% and 88%, respectively. Among total HS cases, 58% were males and 42% were females; 64% were people 65 years old and older, and 36% were younger than 65 years. The average daily occurrence of strokes was 2.22 in the warm season and 2.52 in the cold season, *p* < 0.01 (Supplementary Materials). Among total SAH cases, 37% were males, 63% were females, 66% were elderly, and 34% were non-elderly. From all ICH incident cases, 61% were males, 39% were females, 63% were elderly, and 37% were non-elderly.

Table 2 contains information about the average concentrations and variability of gaseous pollutants and particulate matter monitored, as well as three meteorological variables: temperature, pressure and relative humidity. The 5-year average levels of ambient concentration of SO_2_ during 2014–2018 were 6.31 ± 4.08 μg/m^3^. Higher concentrations were noted for NO_2_, NO and NO_x_: 23.55 ±11.96 μg/m^3^, 22.83 ± 17.57 μg/m^3^, 36.47 ± 29.65 μg/m^3^, respectively. CO concentrations were naturally highest from all gaseous pollutants monitored and were equal to 496.09 ± 203.26 μg/m^3^. Average concentrations of PM_10_ and PM_2.5_ were 26.87 ± 16.68 μg/m^3^ and 20.07 ± 14.27 μg/m^3^, respectively. Seasonality analysis shows high inter-season variations of concentrations of NO_2_, NO, NO_x_, CO, PM_10_ and PM_2.5,_ with statistically higher concentrations during cold seasons (Supplementary Materials). The average temperature was 10.32 °C and ranged from −15.99 °C to 33.08 °C. The average daily relative humidity was 83.05% and ranged from 46.18% to 96.84%. The average atmospheric pressure was 1011.44 hPa and ranged from 978.2 up to 1039.5 hPa.

The Spearman correlation analysis results between air pollutants in Gdansk are presented in Table 3. According to the results, SO_2_ was significantly and positively correlated with NO_2_, NO, NO_x_, PM_2.5_, PM_10_ and CO (*p* < 0.05) but significantly and negatively correlated with O_3_ (*p* < 0.05). The daily average temperature was also negatively correlated with NO_2_ (*p* < 0.05). The correlation of NO_2_, NO and NOx was positive and stronger than that of SO_2_. For example, the correlation of NO_2_ with other pollutants evaluated ranged from 0.75 for PM_10_/PM_2.5_ u to 0.79 for CO. CO correlation with gaseous pollutants as well as particulate matter was positive and significant (*p* < 0.05).

**Figure 1:**
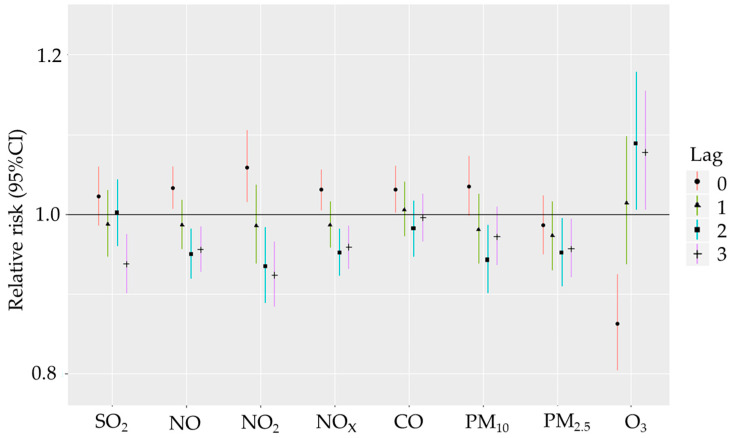
Relative risk with 95% CI of ICH incidence for IQR changes in air pollutant levels (SO_2_—sulphur dioxide, NO—nitrogen oxide, NO_2_—nitrogen dioxide, NO_x_—nitrogen oxides, CO—carbon monoxide, PM_10_—particulate matter with diameter < 10 µm, PM_2.5_—particulate matter with diameter < 2.5 µm, O_3_—ozone) for 0 to 3 days “exposure—disease onset” time delay in single-pollutant model in Gdansk from 1 January 2014 till 31 December 2018 (lag—exposure-outcome delay, e.g., lag1—observed effect is 1 day after exposure).

**Figure 2:**
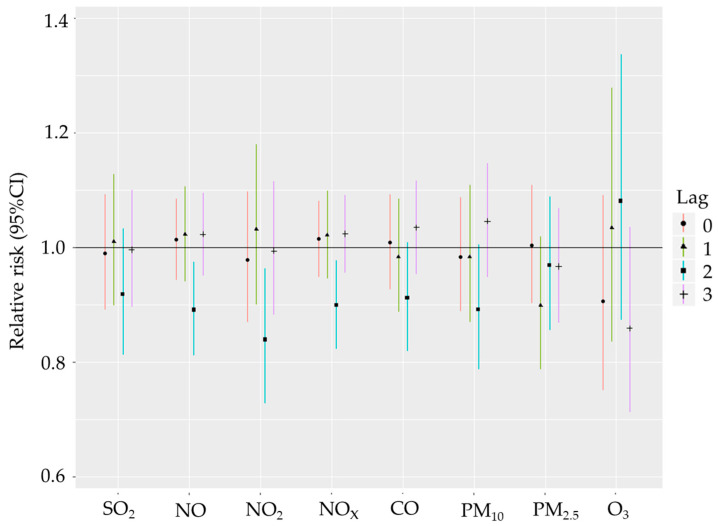
Relative risk with 95% CI of SAH incidence for IQR changes in air pollutant levels (SO_2_—Sulphur dioxide, NO—nitrogen oxide, NO_2_—nitrogen dioxide, NO_x_—nitrogen oxides, CO—carbon monoxide, PM_10_—particulate matter with diameter < 10 µm, PM_2.5_—particulate matter with diameter < 2.5 µm, O_3_—ozone) for 0 to 3 days “exposure—disease onset” time delay in single-pollutant model in Gdansk from 1 January 2014 till 31 December 2018 (lag—exposure-outcome delay, e.g., Lag1—observed effect is 1 day after exposure).

Figure 1 and Figure 2 describe the effects of gaseous pollutants and particulate matter exposure on the daily incidence of all HS and its subtypes in Gdansk from 1 January 2014 to 31 December 2018. The single-day lag model results showed that NO_2_, NO and NO_x_ exposure was associated with increased risk of incident ICH with RR of 1.059 (95% CI: 1.015–1.052 for lag0), 1.033 (95% CI: 1.007–1.060 for lag0) and 1.031 (95% CI: 1.005–1.056 for lag0) respectively. Seemingly, exposure to CO was related to a substantial and statistically significant increase in incidence for ICH stroke with RR: 1.031 (95% CI: 1.002–1.061 for lag0); this relationship will be discussed in depth in the Discussion section. Although not statistically significant, both SO_2_ and PM_10_ were related with notable increases in ICH incidence with RRs equal to 1.023 (95% CI: 0.986–1.060 for lag0) and 1.035 (95% CI: 0.998–1.073 for lag0), respectively. There were no statistically significant positive association between increased risk of incident subarachnoid haemorrhagic stroke (SAH) and any of the ambient air pollutants. Time-delayed response to atmospheric air pollutants was observed for ozone exposure. Interestingly, exposure to ozone within the same day was related to a significant decrease in both ICH and SAH incidence, but within the next days, it caused a sudden and statistically significant increase in both ICH and SAH. This causal relationship was more profound for ICH (Figure 1) than for SAH (Figure 2).

**Figure 3:**
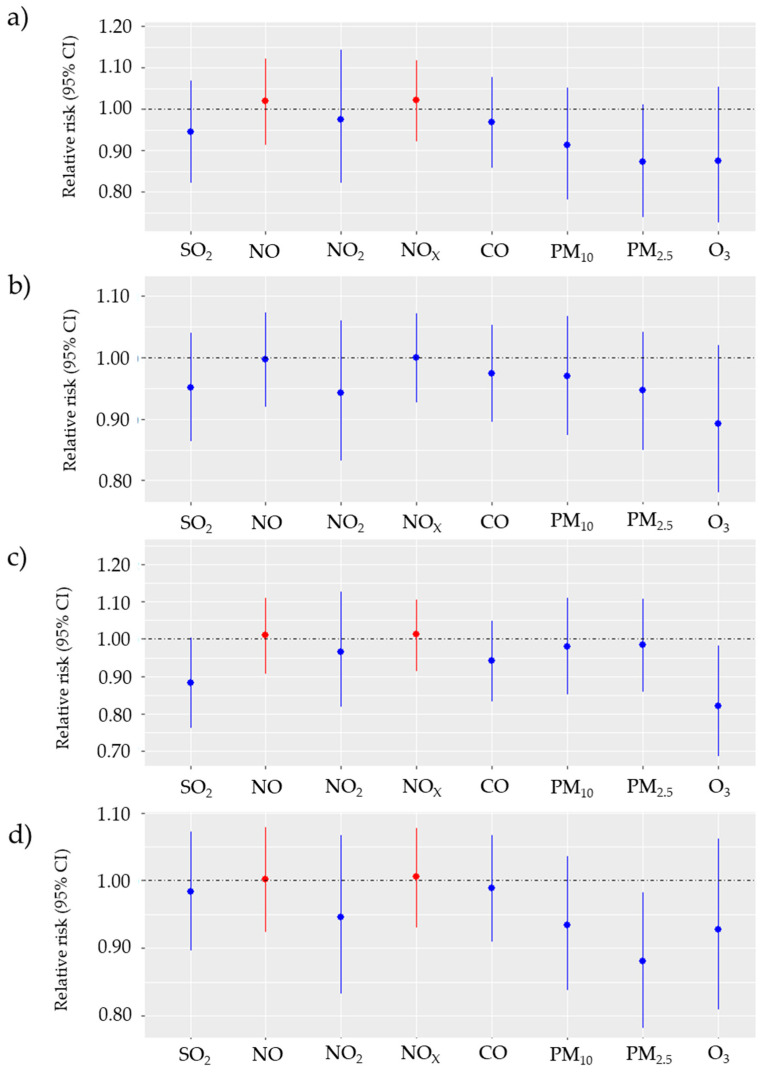
Relative risk with 95% CI of ICH incidence for IQR changes in air pollutant levels SO_2_—Sulphur dioxide, NO—nitrogen oxide, NO_2_—nitrogen dioxide, NO_x_—nitrogen oxides, CO—carbon monoxide, PM_10_—particulate matter with diameter < 10 µm, PM_2.5_—particulate matter with diameter < 2.5 µm, O_3_—ozone) in the single-pollutant model in Gdansk from 1 January 2014 till 31 December 2018 for (**a**) females, (**b**) males, (**c**) population at age 65 and older, (**d**) population younger than 65 years.

**Figure 4:**
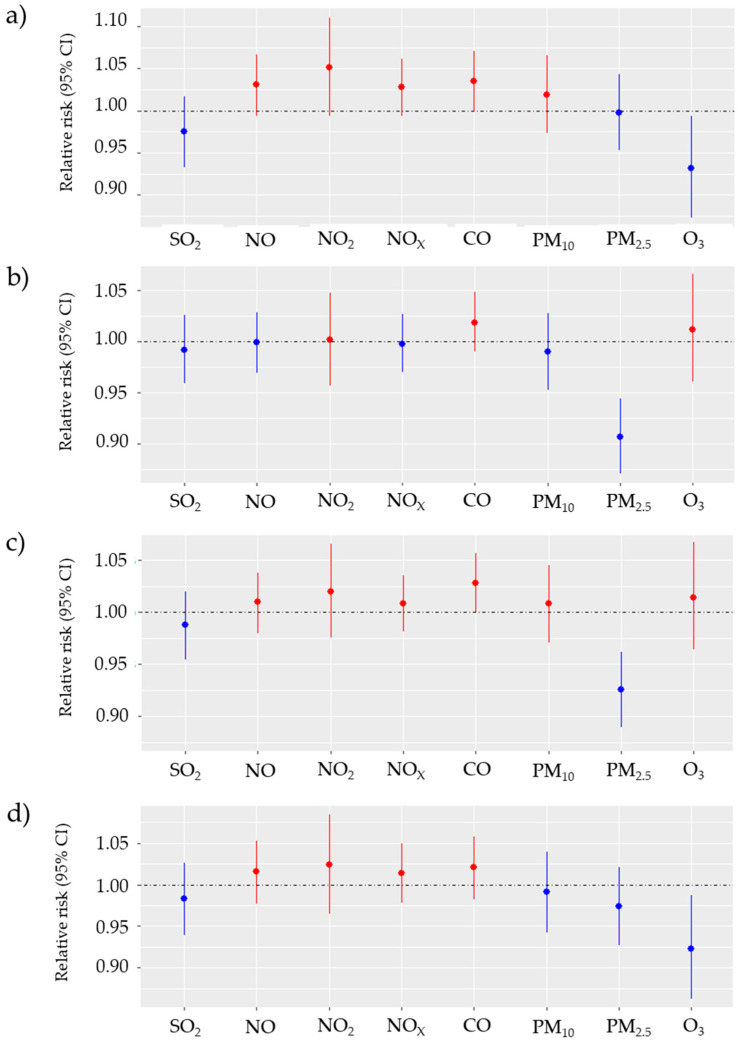
Relative risk with 95% CI of SAH incidence for IQR changes in air pollutant levels SO_2_—Sulphur dioxide, NO—nitrogen oxide, NO_2_—nitrogen dioxide, NO_x_—nitrogen oxides, CO—carbon monoxide, PM_10_—particulate matter with diameter < 10 µm, PM_2.5_—particulate matter with diameter < 2.5 µm, O_3_—ozone) in the single-pollutant model in Gdansk from 1 January 2014 till 31 December 2018 for (**a**) females, (**b**) males, (**c**) population at age 65 and older, (**d**) population younger than 65 years.

Figure 3 and Figure 4 summarise the results of the subgroup analysis. For ICH, changes in NO_2_, NO, NO_x_ and CO concentrations were associated with the risk of incident stroke in females, elderly cases and younger age groups. However, the effect for these subgroups was not statistically significant. This relationship could not be observed for SAH. The effects of NO_2_, NO and NO_x_ exposure on incidents of ICH in females were noticeable and resulted in an increase in RRs: 1.030 (95% CI: 0.998–1.071), 1.049 (95% CI: 0.999–1.115) and 1.026 (95% CI: 0.998–1.070), respectively. Also, the effect of CO for females is worth mentioning, as RR was 1.041 (95% CI: 1.000–1.080). Comparison of vulnerability of elderly and non-elderly groups suggests higher, but not statistically significant, vulnerability of the elderly group towards NO_2_, NO, NO_x_ with RRs equal to 1.019 (95% CI: 0.976–1.055), 1.025 (95% CI: 0.965–1.081) and 1.017 (95% CI: 0.978–1.052), respectively for ICH. No statistically significant associations and strong associations were observed in the subgroups of males and non-elderly cases. 

## 4. Discussion

Compared with ischemic stroke, only a few studies have examined associations between air pollutants and haemorrhagic stroke. Studies undertaken by American, Irish and Chinese scientific groups found no significant associations [37,38,39,40]. However, there exists evidence of a positive correlation between HS and atmospheric air pollution [8,41,42]. 

NO_2_, together with other nitrogen oxides, is one of the main ambient air pollutants. Our study was conducted in an area where air pollution is at a low level. Nonetheless, we have managed to find that low-level ambient air NO_2_ exposure had short-term effects on the incidence of haemorrhagic stroke. These findings are in agreement with a meta-analysis of 6.2 million events across 28 countries [5]. According to the meta-analysis, only NO_2_ was positively associated with haemorrhagic stroke. The study did not provide any proof of the negative effect of the other air pollutants on HS. The same results were obtained by Liu et al., who managed to find a significant association of HS incidence only in relation to NO_2_ on the current day [43]. Our findings are also in line with a multi-city study, which showed that NO_2_ was positively associated with a higher risk of stroke mortality in China, where haemorrhagic stroke accounted for approximately 30% of the total strokes [44]. On the other hand, numerous studies have not found a significant effect of NO_2_ or other nitrogen oxides. In a South London study based on 1758 incident strokes (256 were haemorrhagic), authors found no evidence of an association between either ischemic or haemorrhagic stroke and same-day exposure to PM_10_, O_3_, NO_2_ or NO_x_ [4]. Similar study results were assessed in the Copenhagen study, Denmark. Based on 7485 stroke admissions, among which 687 were haemorrhagic, authors did not observe a significant effect of UFPs, NO_x_ and CO on HS daily admissions [45]. 

Our findings of no or weak association between particulate pollutants (PM_2.5_ and PM_10_) and total HS or stroke subtypes were consistent with a nationwide prospective cohort of postmenopausal women: Women’s Health Initiative [42]. These results are also acknowledged by a meta-analysis reporting that evidence of the association of PM_2.5_ and PM_10_ with hospital admission for total cerebrovascular diseases or ischemic or haemorrhagic stroke was heterogeneous and not statistically significant overall [46]. The Singapore study also showed a lack of a statistically significant relationship between particulate matter exposure and HS incidence [22]. Very recent results of a case-crossover assessment in Boston show no evidence of elevated ICH risk after increases in PM_2.5_ or black carbon [41]. On the other hand, Japanese results of time-series analysis suggest that each 10 μg/m^3^ increase in the previous-day concentration of PM_2.5_ was positively associated with ischemic stroke and intracerebral haemorrhage mortality with a stronger association with subarachnoid haemorrhage mortality [12]. Results from Shanghai show that the incidence of fatal ICH was significantly associated with PM_2.5_ concentration [9]. What is more, the Chinese team observed substantial differences in ORs among subjects with diabetes compared with those without disease. Chiu et al. found that for the single-pollutant model (without adjustment for other pollutants), increased HS admissions were significantly associated with PM_2.5_ levels with an interquartile range rise associated with a 12% and 4% elevation in admissions for HS for warm and cool season respectively [47]. A Portuguese case-crossover study based on 308 patients with spontaneous intracerebral haemorrhage showed a causative relationship with increased PM_2.5_ concentration [8]. What is more, a previously mentioned study of the South London research team found a negative association with PM_10_ suggestive of a 14.6% (95% CI: 0.7–26.5%) fall in risk per 10 µg/m^3^ increase in pollutant [4]. PM_10_ effect on HS was investigated by Han et al. [48]. According to results published by the Korean team, PM_10_ showed positive correlations with intracerebral haemorrhage. Our results, though missing statistical significance, were in line with the results of the Korean study.

Overall, we found significant associations between short-term exposure to ambient carbon monoxide and HS, and these associations were strongest within the same day. Further analysis showed that significance is limited to ICH; as for SAH, this relationship was statistically insignificant. Our results are similar to a previous estimate in the Singapore case-crossover study, where higher levels of CO were significantly associated with an increased risk of HS [22]. However, according to the Singaporean study, the increased risk of HS due to CO exposure persisted for at least 5 days after exposure, whereas in our study, the risk was limited to the same day of exposure. A Danish study reported non-significant associations with HS. In the case-crossover study, ambient carbon monoxide was associated with an increased risk of ischemic stroke, but the effect estimates for HS were statistically insignificant [45]. In a recent meta-analysis of more than 23 million participants, the authors found no significant differences in the association between CO exposure and stroke incidence [49]. According to our study, the more susceptible to CO subgroup would be women and the elderly. These results are in line with time-series analysis in 272 cities in China, where authors observed increased mortality in the female group due to cardiovascular diseases (including strokes) for a 1 mg/m³ increase in average carbon monoxide concentrations on the present day and previous day (lag 0–1) [50]. It needs to be emphasised that CO and NO_2_ have the same source of exposure, namely motor vehicle exhausts, commercial and industrial operations, as well as power stations [51,52]. Thus, the authors reckon that the assessed relationship between HS and CO exposure might be the effect of either actual exposure or the correlation between NO and CO.

Owing to the results of our assessment, the effect of ozone exposure was strongest, positive and statistically significant within a 2- and 3-day lag for total HS. Splitting the effect into two HS subtypes shows that the effect is significant only for ICH, whereas for SAH, this relationship is still positive but not significant. These results are in line with time-stratified bidirectional case-crossover analyses performed for the Boston population [41]. In models stratified by ICH location, associations with ozone remained positive for patients with lobar but not deep ICH. Larger estimates were observed among participants with a probable diagnosis of cerebral amyloid angiopathy. Another research team assessed an increase in the risk of total stroke hospitalisation by 1.9% per interquartile range increase in concentration, but on the current day (lag0) of exposure [53]. Our statement of the significant effect of ozone towards HS incidence stays in accordance with Reykjavik hospital admissions analysis, where daily emergency hospital visits increased by 3.9% per interquartile (IQR) change in average O_3_ the same and two previous days [54]. Contrary to our findings were the results of the Seoul study, where ozone correlated significantly only with subarachnoid haemorrhage. On the other hand, a number of studies did not manage to find any significant relationship between HS and ozone exposure, or the results were ambiguous [49,55,56,57].

Based on our findings, it can be observed that atmospheric pollutants such as NO, NO_2_, NO_x_, CO, particulate matter and ozone have an effect on ICH incidence but not on SAH. It needs to be emphasised that there exists a scarce number of studies that evaluated the effect of atmospheric air pollutants on haemorrhagic stroke incidence or mortality [8,37,38,39,40,42,58]. Even fewer studies differentiate between intracerebral and subarachnoid haemorrhage [4,21,31]. Some studies report no statistical significance between air pollution and haemorrhagic stroke in general [37,39,40]. This finding might suggest that air pollutants exclusively increase intracerebral haemorrhage risk but not subarachnoid haemorrhage. In our opinion, this can be explained by different etiopathogenesis that leads to either to ICH or SAH. The mechanism leading to ICH involves the autonomic respiratory reflex arcs, in which the pollutants trigger an autonomic reflex via pulmonary receptors, baroreceptors, and chemical receptors. This occurrence leads to increased vascular resistance, arrhythmias, and hypertension [59,60]. Non-traumatic subarachnoid haemorrhage is most commonly due to the rupture of a cerebral aneurysm. When an aneurysm ruptures, blood can flow into the subarachnoid space. Other causes of subarachnoid haemorrhage include arteriovenous malformations (AVM), use of blood thinners, head trauma, or idiopathic causes. Many factors have been associated with aneurysmal development and rupture. Such factors include familial/genetic predisposition, cellular aberrations in vascular wall repair or remodelling, and aberrations in local blood flow [61]. Based on the above-mentioned mechanisms, it seems that environmental factors such as atmospheric air pollution may influence ICH incidence more than SAH. At the same time, changes in meteorological conditions, such as temperature and pressure, may affect both ICH and SAH [62,63,64]. 

Our study has some important limitations related to exposure assessment. Since precise addresses of hospitalised patients were not accessible, we used averaged estimates of ambient air pollution from six stations situated at different locations in the Gdansk municipality area. According to Hertwig et al., air quality in cities can be affected by point emissions, chemical and photochemical transformations and the physical state of the atmosphere, which varies spatially [65]. For this reason, we may not fully capture microscale spatial gradients typical of urban environments. Moreover, incidence data came from the National Health Fund. Data derived from large national databases may contain some disease misclassifications and gaps that we do not know about. Differences between the day of stroke symptom onset and the day of hospitalisation may have introduced some degrees of exposure misclassification, which may tend to bias the risk estimates toward the null. However, the majority of patients with haemorrhagic events, due to severe stroke symptoms and regarding stroke care standards, are immediately admitted to stroke units and undergo detailed neuroimaging. Nevertheless, ICD-10 diagnosis was based on registry data and, as such, does not allow for taking into account detailed clinical data, especially comorbidities, which could be regarded as another limitation of our study. Information about comorbidities, as well as taken medications, smoking status and physical activity, would have a great impact on such assessment and would allow a better understanding of the role atmospheric pollution plays in haemorrhagic stroke incidence and mortality. Lastly, our study design has both cross-sectional and ecological characteristics, and for this reason, the inference of the causal relationship between exposure and HS onset might be affected by ecological fallacy. The study limitations have been a matter of broader discussion in our previous publication devoted to ischemic stroke [66].

## 5. Conclusions

To the best of our knowledge, our study is one of the scarce studies that differentiate haemorrhagic strokes into intracerebral (ICH) and subarachnoid (SAH) and is conducted in areas with low-level air pollutants exposure. In contrast to ischemic strokes and myocardial infarctions, haemorrhagic strokes have rarely been a matter of more advanced time-series cross-sectional analyses with respect to exposure to atmospheric pollutants. Especially when it comes to low-level exposures. The results of our study provided evidence that short-time ambient air NO_2_, NO, NO_x_ and CO exposure can adversely influence the incident ICH, even at low pollution levels. 

## Figures and Tables

**Table 1:** Descriptive statistics of daily number of haemorrhagic strokes.

	Count (% of All)	Mean (SD)	Min	Med	Max	IQR
**All haemorrhagic strokes (I60, I61)**	**5181**	**2.84 (2.43)**	**0**	**2**	**14**	**2.5**
Women	2178 (42%)	1.19 (1.29)	0	1	7	2
Men	3003 (58%)	1.65 (1.68)	0	1	9	2
age ≥ 65 years	1877 (36%)	1.03 (1.17)	0	1	6	1
age < 65 years	3304 (64%)	1.81 (1.85)	0	1	9	2
**Subarachnoid haemorrhages (I60)**	**639 (12%)**	**0.35 (0.64)**	**0**	**0**	**3**	**1**
Women	403 (63%)	0.22 (0.53)	0	0	3	0
Men	236 (37%)	0.13 (0.39)	0	0	2	0
age ≥ 65 years	217 (34%)	0.12 (0.38)	0	0	2	0
age < 65 years	422 (66%)	0.23 (0.54)	0	0	3	0
**Intracerebral haemorrhages (I61)**	**4542 (88%)**	**2.49 (2.31)**	**0**	**2**	**13**	**2**
Women	1775 (39%)	0.97 (1.15)	0	1	5	1
Men	2767 (61%)	1.52 (1.62)	0	1	9	2
age ≥ 65 years	1660 (37%)	0.91 (1.10)	0	1	6	1
age < 65 years	2882 (63%)	1.58 (1.75)	0	1	9	2

**Table 2:** Descriptive analysis of pollutant concentration levels and meteorological variables.

Chemical Compounds [μg/m^3^]	Missing Values	Mean (SD)	Min	Max	IQR
SO_2_	0	6.31 (4.08)	1.98	57.73	3.65
NO	0	22.83 (17.57)	4.46	170.94	14.12
NO_2_	0	23.55 (11.96)	5.09	96.49	14.62
NO_x_	0	36.47(29.65)	7.05	294.11	22.52
CO	0	496.09 (203.26)	244.32	2280.12	164.04
PM_10_	0	26.87 (16.68)	5.66	151.17	16.76
PM_2.5_	393	20.07 (14.27)	3.58	178.83	12.84
O_3_	2	55.53 (22.8)	2.36	130	31.7

**Table 3:** Pearson correlation coefficients for air pollutants (*—*p*-value < 0.05).

	SO_2_	NO	NO_2_	NO_X_	CO	PM_10_	PM_2.5_	O_3_
**SO_2_**	1							
**NO**	0.47 *	1						
**NO_2_**	0.56 *	0.88 *	1					
**NO_X_**	0.46 *	1 *	0.86 *	1				
**CO**	0.61 *	0.86 *	0.79 *	0.85 *	1			
**PM_10_**	0.62 *	0.73 *	0.75 *	0.73 *	0.79 *	1		
**PM_2.5_**	0.64 *	0.75 *	0.75 *	0.74 *	0.85 *	0.92 *	1	
**O_3_**	−0.21 *	−0.37 *	−0.26 *	−0.38 *	−0.45 *	−0.2 *	−0.33 *	1

## Data Availability

The data presented in this study are available upon request from the corresponding author.

## Supplementary Materials

The following supporting information can be downloaded at: https://www.mdpi.com/article/10.3390/biomedicines12071562/s1, Table S1. Comparison of mean values of concentration level of air pollutants (t-test) and daily number of haemorrhagic strokes (Mann-Whitney test). Figure S1. Comparison of NO concertation level distribution between summer and heating periods. Figure S2. Comparison of NO_2_ concertation level distribution between summer and heating periods. Figure S3. Comparison of NO_x_ concertation level distribution between summer and heating periods. Figure S4. Comparison of PM_10_ concertation level distribution between summer and heating periods. Figure S5. Comparison of PM_2.5_ concertation level distribution between summer and heating periods. Figure S6. Comparison of CO concertation level distribution between summer and heating periods. Figure S7. Comparison of HS (ICH + SAH) distributions between summer and heating periods.

## References

[1] van Asch C.J., Luitse M.J., Rinkel G.J., van der Tweel I., Algra A., Klijn C.J. (2010). Incidence, Case Fatality, and Functional Outcome of Intracerebral Haemorrhage over Time, according to Age, Sex, and Ethnic Origin: A Systematic Review and Meta-Analysis. Lancet Neurol..

[2] Feigin V.L., Krishnamurthi R.V., Parmar P., Norrving B., Mensah G.A., Bennett D.A., Barker-Collo S., Moran A.E., Sacco R.L., Truelsen T. (2015). Update on the Global Burden of Ischemic and Hemorrhagic Stroke in 1990–2013: The GBD 2013 Study. Neuroepidemiology.

[3] Poon M.T.C., Fonville A.F., Al-Shahi Salman R. (2014). Long-Term Prognosis after Intracerebral Haemorrhage: Systematic Review and Meta-Analysis. J. Neurol. Neurosurg. Psychiatry.

[4] Butland B.K., Atkinson R.W., Crichton S., Barratt B., Beevers S., Spiridou A., Hoang U., Kelly F.J., Wolfe C.D. (2017). Air Pollution and the Incidence of Ischaemic and Haemorrhagic Stroke in the South London Stroke Register: A Case–Cross-over Analysis. J. Epidemiol. Community Health.

[5] Shah A.S.V., Lee K.K., McAllister D.A., Hunter A., Nair H., Whiteley W., Langrish J.P., Newby D.E., Mills N.L. (2015). Short Term Exposure to Air Pollution and Stroke: Systematic Review and Meta-Analysis. BMJ.

[6] Chan C.-C., Chuang K.-J., Chien L.-C., Chen W.-J., Chang W.-T. (2006). Urban Air Pollution and Emergency Admissions for Cerebrovascular Diseases in Taipei, Taiwan. Eur. Heart J..

[7] Zhang C., Ding R., Xiao C., Xu Y., Cheng H., Zhu F., Lei R., Di D., Zhao Q., Cao J. (2017). Association between Air Pollution and Cardiovascular Mortality in Hefei, China: A Time-Series Analysis. Environ. Pollut..

[8] Nzwalo H., Guilherme P., Nogueira J., Félix C., André A., Teles J., Mouzinho M., Ferreira F., Marreiros A., Logallo N. (2019). Fine Particulate Air Pollution and Occurrence of Spontaneous Intracerebral Hemorrhage in an Area of Low Air Pollution. Clin. Neurol. Neurosurg..

[9] Qian Y., Yu H., Cai B., Fang B., Wang C. (2019). Association between Incidence of Fatal Intracerebral Hemorrhagic Stroke and Fine Particulate Air Pollution. Environ. Health Prev. Med..

[10] Yorifuji T., Kawachi I., Sakamoto T., Doi H. (2011). Associations of Outdoor Air Pollution with Hemorrhagic Stroke Mortality. J. Occup. Environ. Med..

[11] Chien T.-Y., Ting H.-W., Chan C.-L., Yang N.-P., Pan R.-H., Lai K.R., Hung S.-I. (2017). Does the Short-Term Effect of Air Pollution Influence the Incidence of Spontaneous Intracerebral Hemorrhage in Different Patient Groups? Big Data Analysis in Taiwan. Int. J. Environ. Res. Public Health.

[12] Jun Rui M.L., Tan J., Tan B.Y.-Q., Yeo T.T., Sharma V.K. (2022). Air Pollution and Intracranial Hemorrhage. Ann. Indian Acad. Neurol..

[13] Tykhonova S., Shtanko V., Khyzhnyak O., Tofan N. (2022). The Effect of Pollution on Hypertension and on the Total Risk Score in Hypertensive Patients. E J. Cardiol. Pract..

[14] Choi Y.-J., Kim S.-H., Kang S.-H., Kim S.-Y., Kim O.-J., Yoon C.-H., Lee H.-Y., Youn T.-J., Chae I.-H., Kim C.-H. (2019). Short-Term Effects of Air Pollution on Blood Pressure. Sci. Rep..

[15] Brook R.D., Rajagopalan S. (2020). Inhaling Hypertension. Hypertension.

[16] Camilli M., Russo M., Rinaldi R., Caffè A., La Vecchia G., Bonanni A., Iannaccone G., Basile M., Vergallo R., Aurigemma C. (2022). Air Pollution and Coronary Vasomotor Disorders in Patients with Myocardial Ischemia and Unobstructed Coronary Arteries. J. Am. Coll. Cardiol..

[17] Di Q., Wang Y., Zanobetti A., Wang Y., Koutrakis P., Choirat C., Dominici F., Schwartz J.D. (2017). Air Pollution and Mortality in the Medicare Population. N. Engl. J. Med..

[18] Crowley R.A., Moyer D.V., DeLong D.M. (2016). Climate Change and Health. Ann. Intern. Med..

[19] Cheong K.H., Ngiam N.J., Morgan G.G., Pek P.P., Tan B.Y.-Q., Lai J.W., Koh J.M., Ong M.E.H., Ho A.F.W. (2019). Acute Health Impacts of the Southeast Asian Transboundary Haze Problem—A Review. Int. J. Environ. Res. Public Health.

[20] Ljungman P.L., Mittleman M.A. (2014). Ambient Air Pollution and Stroke. Stroke.

[21] Verhoeven J.I., Allach Y., Vaartjes I.C.H., Klijn C.J.M., de Leeuw F.-E. (2021). Ambient Air Pollution and the Risk of Ischaemic and Haemorrhagic Stroke. Lancet Planet. Health.

[22] Ho A.F.W., Lim M.J.R., Zheng H., Leow A.S.-T., Tan B.Y.-Q., Pek P.P., Raju Y., Seow W.-J., Yeo T.T., Sharma V.K. (2022). Association of Ambient Air Pollution with Risk of Hemorrhagic Stroke: A Time-Stratified Case Crossover Analysis of the Singapore Stroke Registry. Int. J. Hyg. Environ. Health.

[23] Dąbrowiecki P., Chciałowski A., Dąbrowiecka A., Badyda A. (2022). Ambient Air Pollution and Risk of Admission due to Asthma in the Three Largest Urban Agglomerations in Poland: A Time-Stratified, Case-Crossover Study. Int. J. Environ. Res. Public Health.

[24] Dąbrowiecki P., Badyda A., Chciałowski A., Czechowski P.O., Wrotek A. (2022). Influence of Selected Air Pollutants on Mortality and Pneumonia Burden in Three Polish Cities over the Years 2011–2018. J. Clin. Med..

[25] Dąbrowiecki P., Chciałowski A., Dąbrowiecka A., Piórkowska A., Badyda A. (2023). Air Pollution and Long-Term Risk of Hospital Admission due to Chronic Obstructive Pulmonary Disease Exacerbations in Poland: A Time-Stratified, Case-Crossover Study. Pol. Arch. Med. Wewnętrznej.

[26] Dąbrowiecki P., Chciałowski A., Dąbrowiecka A., Piórkowska A., Badyda A. (2023). Exposure to Ambient Air Pollutants and Short-Term Risk for Exacerbations of Allergic Rhinitis: A Time-Stratified, Case-Crossover Study in the Three Largest Urban Agglomerations in Poland. Respir. Physiol. Neurobiol..

[27] Gdansk Climate: Weather by Month, Temperature, Precipitation, When to Go. https://www.climatestotravel.com/climate/poland/gdansk.

[28] Raporty o Stanie Środowiska. https://www.gios.gov.pl/images/dokumenty/pms/raporty/stan_srodowiska_2020_pomorskie.pdf.

[29] WHO Definition of an Older or Elderly Person|PDF|Ageing|Old Age. https://www.scribd.com/document/190077600/WHO-Definition-of-an-Older-or-Elderly-Person.

[30] Rubin D.B. (2004). Multiple Imputation for Nonresponse in Surveys.

[31] Li J., Huang J., Wang Y., Yin P., Wang L., Liu Y., Pan X., Zhou M., Li G. (2020). Years of Life Lost from Ischaemic and Haemorrhagic Stroke Related to Ambient Nitrogen Dioxide Exposure: A Multicity Study in China. Ecotoxicol. Environ. Saf..

[32] Wolf K., Hoffmann B., Andersen Z.J., Atkinson R.W., Bauwelinck M., Bellander T., Brandt J., Brunekreef B., Cesaroni G., Chen J. (2021). Long-Term Exposure to Low-Level Ambient Air Pollution and Incidence of Stroke and Coronary Heart Disease: A Pooled Analysis of Six European Cohorts within the ELAPSE Project. Lancet Planet. Health.

[33] Chen Z., Wang B., Hu Y., Dai L., Liu Y., Wang J., Cao X., Wu Y., Zhou T., Cui X. (2022). Short-Term Effects of Low-Level Ambient Air NO_2_ on the Risk of Incident Stroke in Enshi City, China. Int. J. Environ. Res. Public Health.

[34] Yin P., Chen R., Wang L., Meng X., Liu C., Niu Y., Lin Z., Liu Y., Liu J., Qi J. (2017). Ambient Ozone Pollution and Daily Mortality: A Nationwide Study in 272 Chinese Cities. Environ. Health Perspect..

[35] Wang F., Liu H., Li H., Liu J., Guo X., Yuan J., Hu Y., Wang J., Lu L. (2018). Ambient Concentrations of Particulate Matter and Hospitalization for Depression in 26 Chinese Cities: A Case-Crossover Study. Environ. Int..

[36] Wang L., Liu C., Meng X., Niu Y., Lin Z., Liu Y., Liu J., Qi J., You J., Tse L.A. (2018). Associations between Short-Term Exposure to Ambient Sulfur Dioxide and Increased Cause-Specific Mortality in 272 Chinese Cities. Environ. Int..

[37] Fisher J.A., Puett R.C., Laden F., Wellenius G.A., Sapkota A., Liao D., Yanosky J.D., Carter-Pokras O., He X., Hart J.E. (2019). Case-Crossover Analysis of Short-Term Particulate Matter Exposures and Stroke in the Health Professionals Follow-up Study. Environ. Int..

[38] Tian Y., Liu H., Wu Y., Si Y., Song J., Cao Y., Li M., Wu Y., Wang X., Chen L. (2019). Association between Ambient Fine Particulate Pollution and Hospital Admissions for Cause Specific Cardiovascular Disease: Time Series Study in 184 Major Chinese Cities. BMJ.

[39] Byrne C.P., Bennett K.E., Hickey A., Kavanagh P., Broderick B., O’Mahony M., Williams D.J. (2020). Short-Term Air Pollution as a Risk for Stroke Admission: A Time-Series Analysis. Cerebrovasc. Dis..

[40] Gu J., Shi Y., Chen N., Wang H., Chen T. (2020). Ambient Fine Particulate Matter and Hospital Admissions for Ischemic and Hemorrhagic Strokes and Transient Ischemic Attack in 248 Chinese Cities. Sci. Total Environ..

[41] Wilker E.H., Mostofsky E., Fossa A., Koutrakis P., Warren A., Charidimou A., Mittleman M.A., Viswanathan A. (2018). Ambient Pollutants and Spontaneous Intracerebral Hemorrhage in Greater Boston. Stroke.

[42] Sun S., Stewart J.D., Eliot M.N., Yanosky J.D., Liao D., Tinker L.F., Eaton C.B., Whitsel E.A., Wellenius G.A. (2019). Short-Term Exposure to Air Pollution and Incidence of Stroke in the Women’s Health Initiative. Environ. Int..

[43] Liu H., Tian Y., Xu Y., Huang Z., Huang C., Hu Y., Zhang J. (2017). Association between Ambient Air Pollution and Hospitalization for Ischemic and Hemorrhagic Stroke in China: A Multicity Case-Crossover Study. Environ. Pollut..

[44] Chen R., Zhang Y., Yang C., Zhao Z., Xu X., Kan H. (2013). Acute Effect of Ambient Air Pollution on Stroke Mortality in the China Air Pollution and Health Effects Study. Stroke.

[45] Andersen Z.J., Olsen T.S., Andersen K.K., Loft S., Ketzel M., Raaschou-Nielsen O. (2010). Association between Short-Term Exposure to Ultrafine Particles and Hospital Admissions for Stroke in Copenhagen, Denmark. Eur. Heart J..

[46] Wang Y., Eliot M.N., Wellenius G.A. (2014). Short-Term Changes in Ambient Particulate Matter and Risk of Stroke: A Systematic Review and Meta-Analysis. J. Am. Heart Assoc..

[47] Chiu H.-F., Chang C.-C., Yang C.-Y. (2014). Relationship Between Hemorrhagic Stroke Hospitalization and Exposure to Fine Particulate Air Pollution in Taipei, Taiwan. J. Toxicol. Environ. Health Part A.

[48] Han M.-H., Yi H.-J., Ko Y., Kim Y.-S., Lee Y.-J. (2016). Association between Hemorrhagic Stroke Occurrence and Meteorological Factors and Pollutants. BMC Neurol..

[49] Niu Z., Liu F., Yu H., Wu S., Xiang H. (2021). Association between Exposure to Ambient Air Pollution and Hospital Admission, Incidence, and Mortality of Stroke: An Updated Systematic Review and Meta-Analysis of More than 23 Million Participants. Environ. Health Prev. Med..

[50] Liu C., Yin P., Chen R., Meng X., Wang L., Niu Y., Lin Z., Liu Y., Liu J., Qi J. (2018). Ambient Carbon Monoxide and Cardiovascular Mortality: A Nationwide Time-Series Analysis in 272 Cities in China. Lancet Planet. Health.

[51] Dahmann D., Morfeld P., Monz C., Noll B., Gast F. (2009). Exposure Assessment for Nitrogen Oxides and Carbon Monoxide in German Hard Coal Mining. Int. Arch. Occup. Environ. Health.

[52] Chen T.-M., Kuschner W.G., Gokhale J., Shofer S. (2007). Outdoor Air Pollution: Nitrogen Dioxide, Sulfur Dioxide, and Carbon Monoxide Health Effects. Am. J. Med. Sci..

[53] Xu J., Geng W., Geng X., Cui L., Ding T., Xiao C., Zhang J., Tang J., Zhai J. (2020). Study on the Association between Ambient Air Pollution and Daily Cardiovascular Death in Hefei, China. Environ. Sci. Pollut. Res. Int..

[54] Carlsen H.K., Forsberg B., Meister K., Gíslason T., Oudin A. (2013). Ozone Is Associated with Cardiopulmonary and Stroke Emergency Hospital Visits in Reykjavík, Iceland 2003–2009. Environ. Health.

[55] Atkinson R.W., Butland B.K., Dimitroulopoulou C., Heal M.R., Stedman J.R., Carslaw N., Jarvis D., Heaviside C., Vardoulakis S., Walton H. (2016). Long-Term Exposure to Ambient Ozone and Mortality: A Quantitative Systematic Review and Meta-Analysis of Evidence from Cohort Studies. BMJ Open.

[56] Montresor-López J.A., Yanosky J.D., Mittleman M.A., Sapkota A., He X., Hibbert J.D., Wirth M.D., Puett R.C. (2016). Short-Term Exposure to Ambient Ozone and Stroke Hospital Admission: A Case-Crossover Analysis. J. Expo. Sci. Environ. Epidemiol..

[57] Liu S., Zhang Y., Ma R., Liu X., Liang J., Lin H., Shen P., Zhang J., Lu P., Tang X. (2022). Long-Term Exposure to Ozone and Cardiovascular Mortality in a Large Chinese Cohort. Environ. Int..

[58] Tian Y., Liu H., Zhao Z., Xiang X., Li M., Juan J., Song J., Cao Y., Wang X., Chen L. (2018). Association between Ambient Air Pollution and Daily Hospital Admissions for Ischemic Stroke: A Nationwide Time-Series Analysis. PLoS Med..

[59] Hahad O., Lelieveld J., Birklein F., Lieb K., Daiber A., Münzel T. (2020). Ambient Air Pollution Increases the Risk of Cerebrovascular and Neuropsychiatric Disorders through Induction of Inflammation and Oxidative Stress. Int. J. Mol. Sci..

[60] Perez C.M., Hazari M.S., Farraj A.K. (2015). Role of Autonomic Reflex Arcs in Cardiovascular Responses to Air Pollution Exposure. Cardiovasc. Toxicol..

[61] Hackman J.L., Nelson A.M., Ma O.J., Tintinalli J.E., Stapczynski J.S., Ma O.J., Yealy D.M., Meckler G.D., Cline D.M. (2016). Spontaneous Subarachnoid and Intracerebral Hemorrhage. Tintinalli’s Emergency Medicine: A Comprehensive Study Guide.

[62] Vasquez H.E., Prasad L., Moscote-Salazar L.R., Agrawal A. (2021). Atmospheric Variables and Subarachnoid Hemorrhage: Narrative Review. Egypt. J. Neurosurg..

[63] Wang X., Cao Y., Hong D., Zheng D., Richtering S., Sandset E.C., Leong T.H., Arima H., Islam S., Salam A. (2016). Ambient Temperature and Stroke Occurrence: A Systematic Review and Meta-Analysis. Int. J. Environ. Res. Public Health.

[64] Guo P., Wang Y., Feng W., Wu J., Fu C., Deng H., Huang J., Wang L., Zheng M., Liu H. (2017). Ambient Air Pollution and Risk for Ischemic Stroke: A Short-Term Exposure Assessment in South China. Int. J. Environ. Res. Public Health.

[65] Hertwig D., Grimmond S., Kotthaus S., Vanderwel C., Gough H., Haeffelin M., Robins A. (2021). Variability of Physical Meteorology in Urban Areas at Different Scales: Implications for Air Quality. Faraday Discuss..

[66] Czernych R., Badyda A.J., Kozera G., Zagożdżon P. (2023). Assessment of Low-Level Air Pollution and Cardiovascular Incidence in Gdansk, Poland: Time-Series Cross-Sectional Analysis. J. Clin. Med..

