# Supplementary material for: Air Pollution Increases Risk of Occurrence of Intracerebral Haemorrhage but Not of Subarachnoid Haemorrhage: Time-Series Cross-Sectional Study
Source: Biomedicines. 2024 Jul 15;12(7):1562. doi: 10.3390/biomedicines12071562 (PMC11274972; doi:10.3390/biomedicines12071562)
Supplement: Supplementary file 1 [file biomedicines-12-01562-s001.zip › biomedicines-3069088-supplementary.pdf]

SUPPLEMEMTARY MATERIALS

Table S1. Comparison of mean values of concentration level of air pollutants (t-test) and daily number of haemorrhagic strokes (Mann-Whitney test)

	Summer period	Heating Period	p-value
**Environmental variables**			
**NO**	18.45	27.28	<0,001
**NO_2**	20.15	27.00	<0,001
**NO_X**	29.22	43.84	<0,001
**PM_{10}**	23.00	30.81	<0,001
**$PM_{2.5}$**	15.94	24.13	<0,001
**CO**	412.53	580.96	<0,001
**Hemorrhagic strokes**	2.22	2.51	0.002*

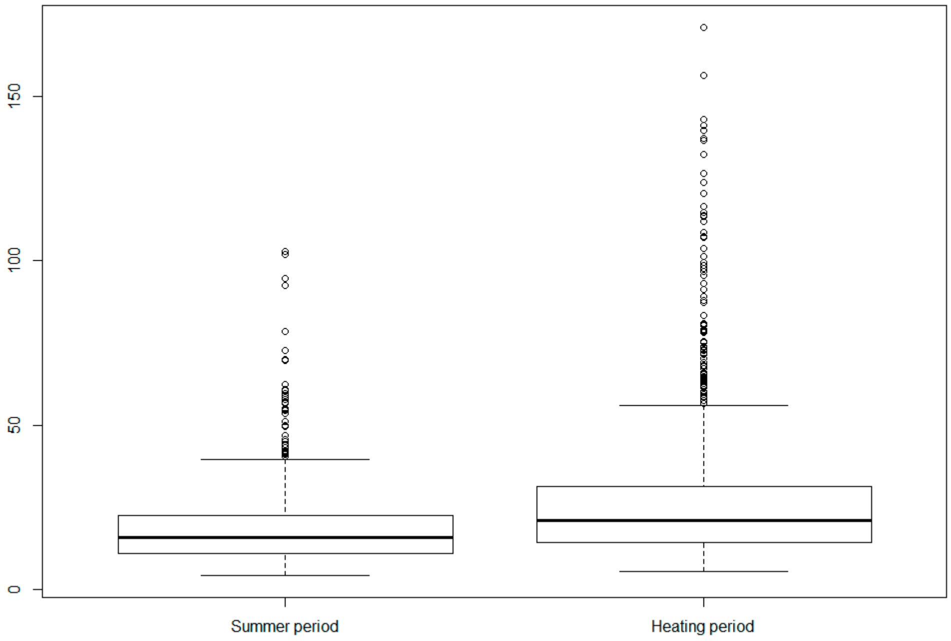

Figure S1. Comparison of NO concertation level distribution between summer and heating periods

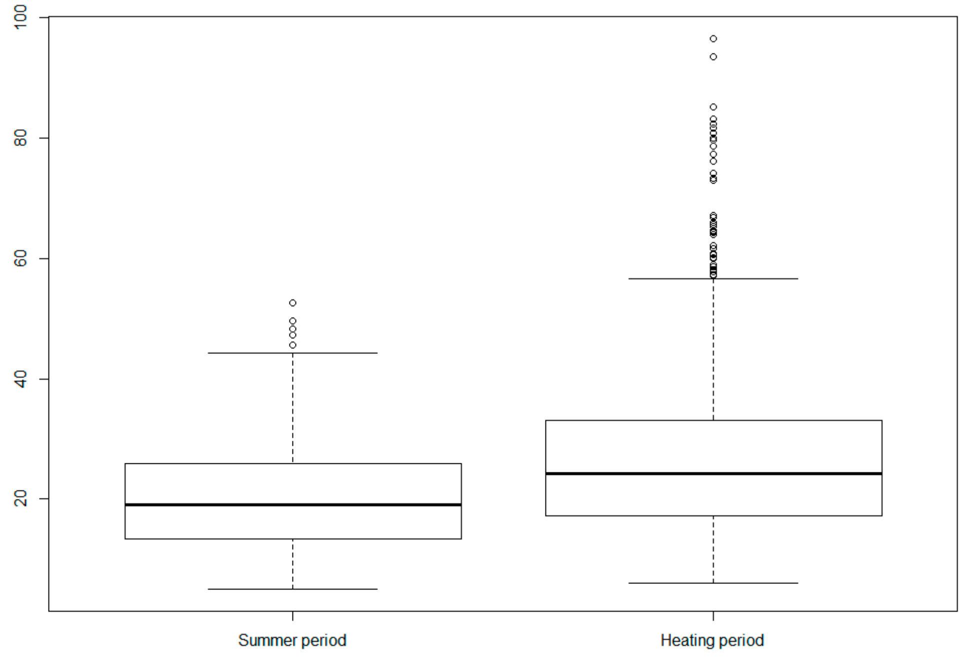

Figure S2. Comparison of NO_2 concertation level distribution between summer and heating periods

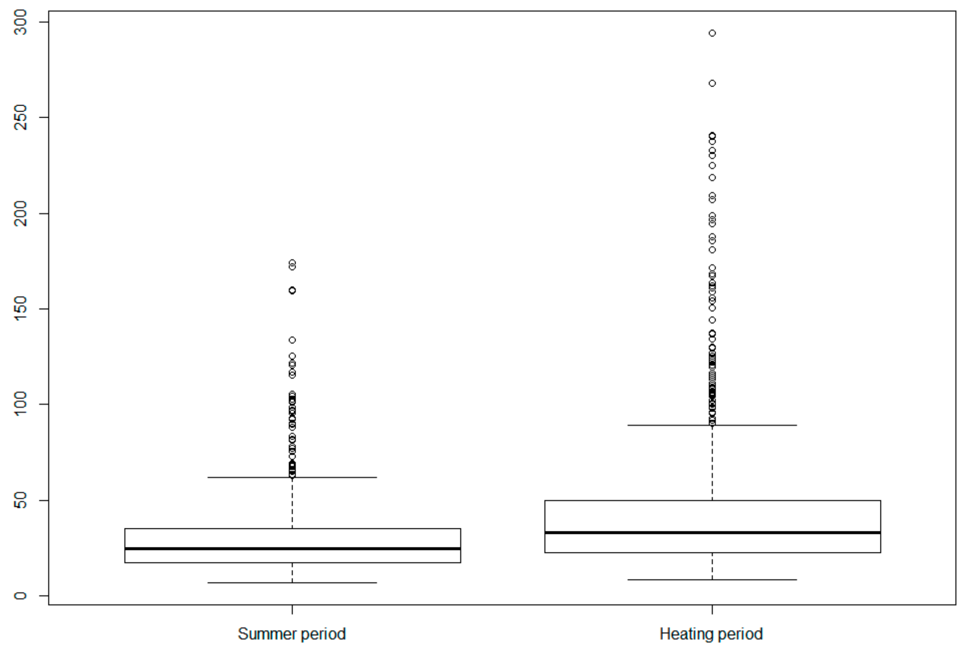

Figure S3. Comparison of NO_x concertation level distribution between summer and heating periods

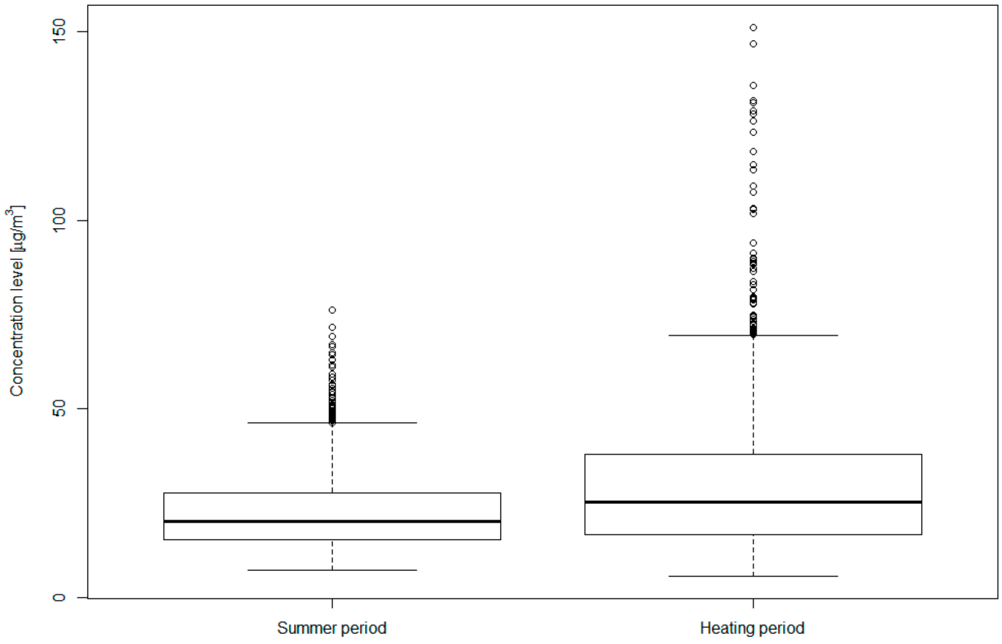

Figure S4. Comparison of PM_{10} concertation level distribution between summer and heating periods

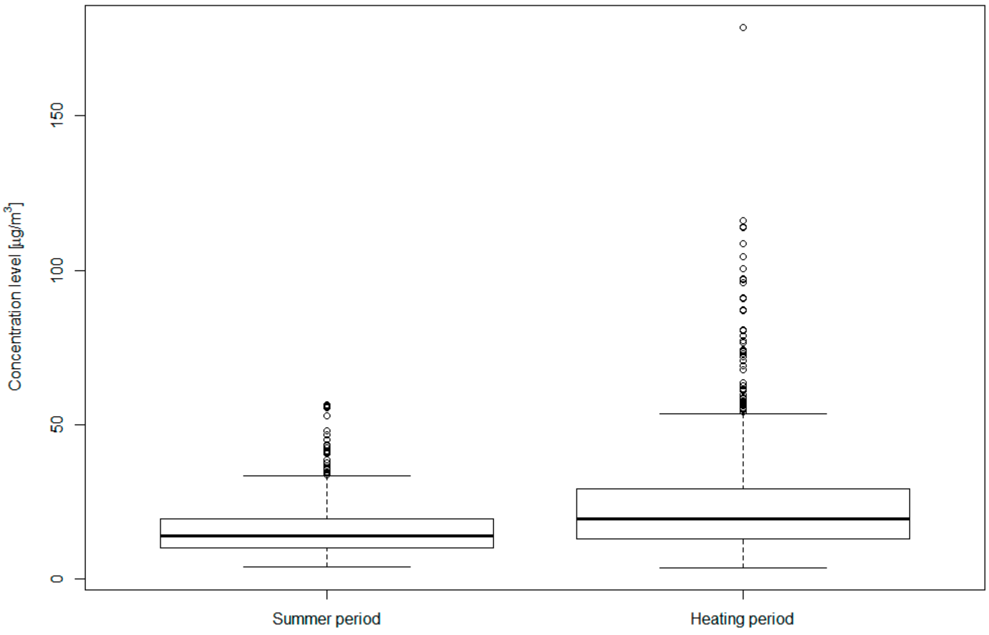

Figure S5. Comparison of $PM_{2.5}$ concertation level distribution between summer and heating periods

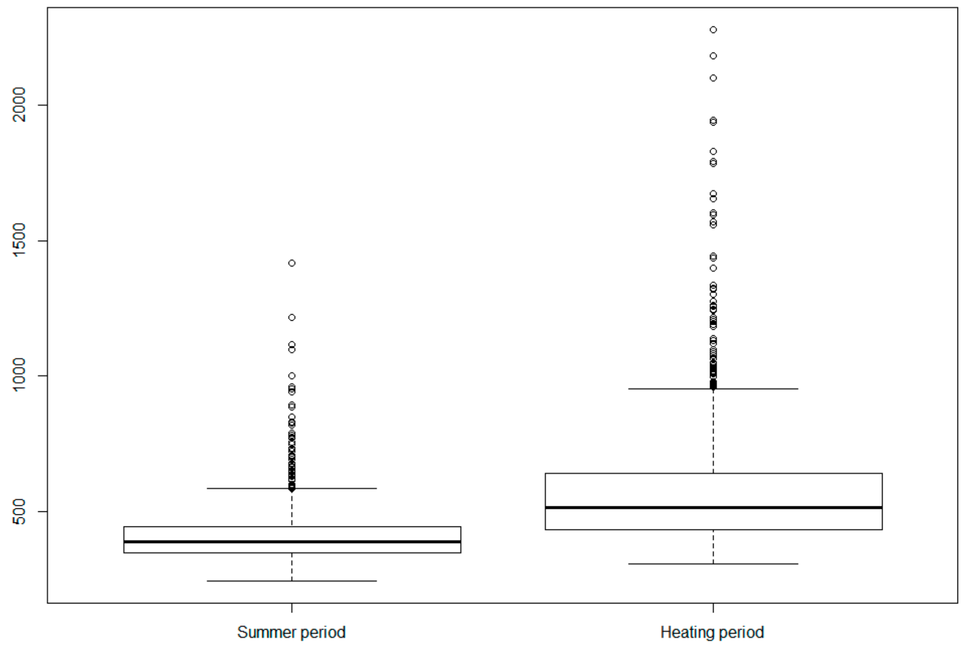

Figure S6. Comparison of CO concertation level distribution between summer and heating periods

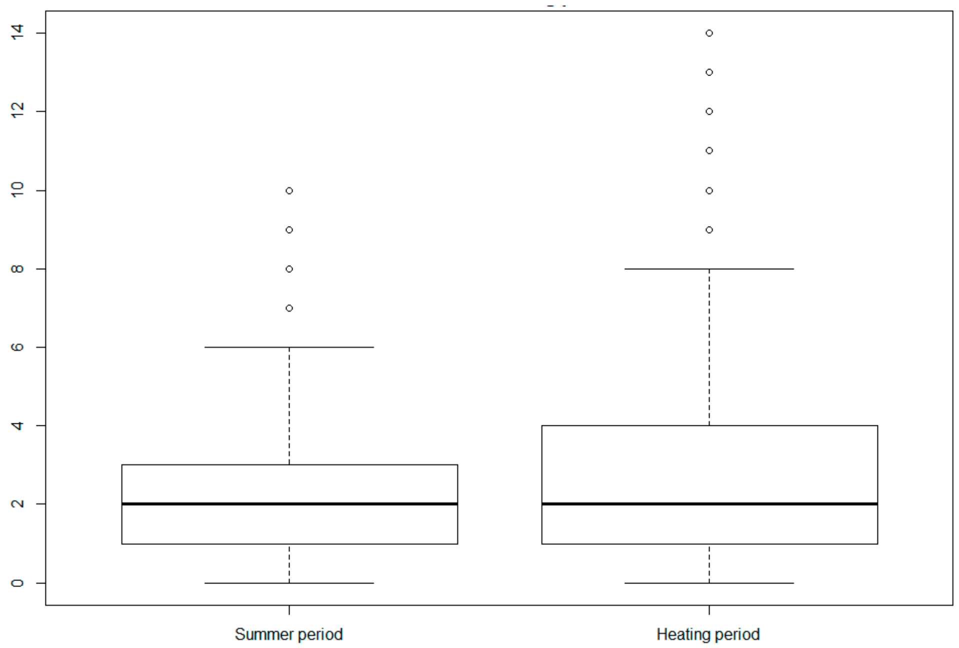

Figure S7. Comparison of HS (ICH + SAH) distributions between summer and heating periods
